# Magnetic resonance image-based brain tumour segmentation methods: A systematic review

**DOI:** 10.1177/20552076221074122

**Published:** 2022-03-16

**Authors:** Jayendra M Bhalodiya, Sarah N Lim Choi Keung, Theodoros N Arvanitis

**Affiliations:** 1 Institute of Digital Healthcare, 120959 Warwick Manufacturing Group, 2707 The University of Warwick, UK; 2 School of Engineering and Applied Science, Ahmedabad University, Gujarat, India

**Keywords:** Brain tumour, magnetic resonance imaging, segmentation, systematic review, brain, artificial intelligence

## Abstract

**Background:**

Image segmentation is an essential step in the analysis and subsequent characterisation of brain tumours through magnetic resonance imaging. In the literature, segmentation methods are empowered by open-access magnetic resonance imaging datasets, such as the brain tumour segmentation dataset. Moreover, with the increased use of artificial intelligence methods in medical imaging, access to larger data repositories has become vital in method development.

**Purpose:**

To determine what automated brain tumour segmentation techniques can medical imaging specialists and clinicians use to identify tumour components, compared to manual segmentation.

**Methods:**

We conducted a systematic review of 572 brain tumour segmentation studies during 2015–2020. We reviewed segmentation techniques using T1-weighted, T2-weighted, gadolinium-enhanced T1-weighted, fluid-attenuated inversion recovery, diffusion-weighted and perfusion-weighted magnetic resonance imaging sequences. Moreover, we assessed physics or mathematics-based methods, deep learning methods, and software-based or semi-automatic methods, as applied to magnetic resonance imaging techniques. Particularly, we synthesised each method as per the utilised magnetic resonance imaging sequences, study population, technical approach (such as deep learning) and performance score measures (such as Dice score).

**Statistical tests:**

We compared median Dice score in segmenting the whole tumour, tumour core and enhanced tumour.

**Results:**

We found that T1-weighted, gadolinium-enhanced T1-weighted, T2-weighted and fluid-attenuated inversion recovery magnetic resonance imaging are used the most in various segmentation algorithms. However, there is limited use of perfusion-weighted and diffusion-weighted magnetic resonance imaging. Moreover, we found that the U-Net deep learning technology is cited the most, and has high accuracy (Dice score 0.9) for magnetic resonance imaging-based brain tumour segmentation.

**Conclusion:**

U-Net is a promising deep learning technology for magnetic resonance imaging-based brain tumour segmentation. The community should be encouraged to contribute open-access datasets so training, testing and validation of deep learning algorithms can be improved, particularly for diffusion- and perfusion-weighted magnetic resonance imaging, where there are limited datasets available.

## Introduction

Brain tumours are malignancies of brain tissues. Characterising such tissues and identifying related genes can help to estimate tumour spread, and further help to identify tumour grades for the treatment planning.^1,2^ Such characterisation comprises of the different tumour components assessment. Components such as tumour core, boundary of tumour core, peritumoral oedema, cellular proliferation (an increase of the number of cells), cellular infiltration (migration of cells or excessive growth) and vascular proliferation (leaky blood vessels) are of great clinical interest.^1,3^ Current practice involves various magnetic resonance imaging (MRI) approaches to visualise these tumour components.^4^ Particularly, the tumour core can be visible in T2-weighted MRI, and T1-weighted MRI, the enhanced boundary of tumour core can be seen in gadolinium-based T1-weighted MRI (T1-Gd), peritumoral oedema can be visible in fluid-attenuated inversion recovery (FLAIR) MRI, while cellular proliferation, cellular infiltration and vascular proliferation can be visualised in diffusion-weighted MRI and perfusion-weighted MRI.

In the literature, MRI-based image processing methods addressed the outlining of tumour components.^5^ These methods can distinguish between healthy and tumour tissues. Moreover, they can distinguish among different tumour components within the tumour. Such methods are commonly known as segmentation methods,^5^ which can be manual, semi-automatic or automatic. With the increase of segmentation methods, the medical image analysis community has reviewed them using publicly available benchmark datasets (e.g. the brain tumour segmentation – BraTS dataset) to assess their performance.^5,6^ Additionally, individual reviews addressing only deep learning methods,^7^ only automated methods,^8^ classical reviews,^9^ and practical implications^10^ are reported. However, a systematic review to identify promising, and widely adapted brain tumor segmentation methods is not reported in the literature. Moreover, in the literature, individual methods, and their validation show technical advancements in most MR image-based approaches, but they are not fully explored in all MRI sequences in the imaging of brain tumours. For example, diffusion-weighted, and perfusion-weighted MRI are often overlooked or limited in brain tumor segmentation method development, and validation. In addition, a recent trend of deep learning methods has extensively contributed to the development of automatic segmentation methods, in order to avoid the subjective, and time-consuming nature of manual techniques. However, the performance of these deep learning methods relies on the data types used, single or multi-centre data collection, the number of available data samples for training, validation and testing of the approach. Therefore, it is crucial to explore the various studies in deep learning methods. Such a study can help understanding and justifying the need for further large open-access data repositories and alternative artificial intelligence (AI)-based techniques, such as transfer learning.

In this systematic review, we addressed the following PICO (P: population, I: intervention, C: comparison, O: outcome) format research question:

What automated brain tumour segmentation techniques can medical imaging specialists, and clinicians use to identify tumour components, compared to manual segmentation?

To answer the above question, we reviewed brain tumour segmentation methods which are based on physics or mathematics models, deep learning models, and software or semi-automatic methods. The methods, which use at least four types of MRI sequences (T1-weighted, T1-Gd, T2-weighted and FLAIR MRI), are included in the synthesis as they are common in clinical practice. Articles based on images from other MRI sequences are discussed, individually. The accuracy measures and study population of the various segmentation methods are also reviewed. Moreover, deep learning architectures are reviewed for their underlying network architecture (for example, U-Net, VGG etc.) with a list of articles that adapted such architectures in their studies. Technical specifications of such architectures are listed in the discussion.

## Materials and methods

### Study protocol

A protocol for this study is prepared internally but not registered elsewhere. However, the PROSPERO database is checked to ensure the originality of the study.

### Article search

For source articles’ systematic identification, we have searched the following online databases: PubMed, Embase Ovid and Engineering Village. The different combinations of the keywords ‘glioma’, ‘medulloblastoma’, ‘brain tumour’ and ‘segmentation’ were used. After discussion among the authors, the combination, ‘glioma’, and ‘segmentation’, and ‘brain tumour’, was used for the article search. The search duration was defined to include articles published in the period from 2015 until 2020. In PubMed, the specific search filters were full-text, humans, English and segmentation keyword must be in the article title or abstract. In Engineering Village, the specific search filters were that the segmentation keyword must be in the abstract, and glioma, and brain tumour keywords must be in the subject or title or abstract. All the articles were stored using Zotero^11^ software. After removing the duplicates, study inclusion/exclusion criteria were applied.

### Study inclusion/exclusion criteria

Articles are screened at two levels for inclusion/exclusion. First, the articles are screened at the abstract, and title level. At this level, the studies, which are not segmentation studies, are excluded. The excluded studies are grouped into the following categories: clinical analysis studies, case studies, image pre-processing studies, general overall surveys, tumour classification studies, tumour detection and identification studies, information learning for model training studies, surgical planning studies, datasets and not accessible studies.

**Figure 1.**
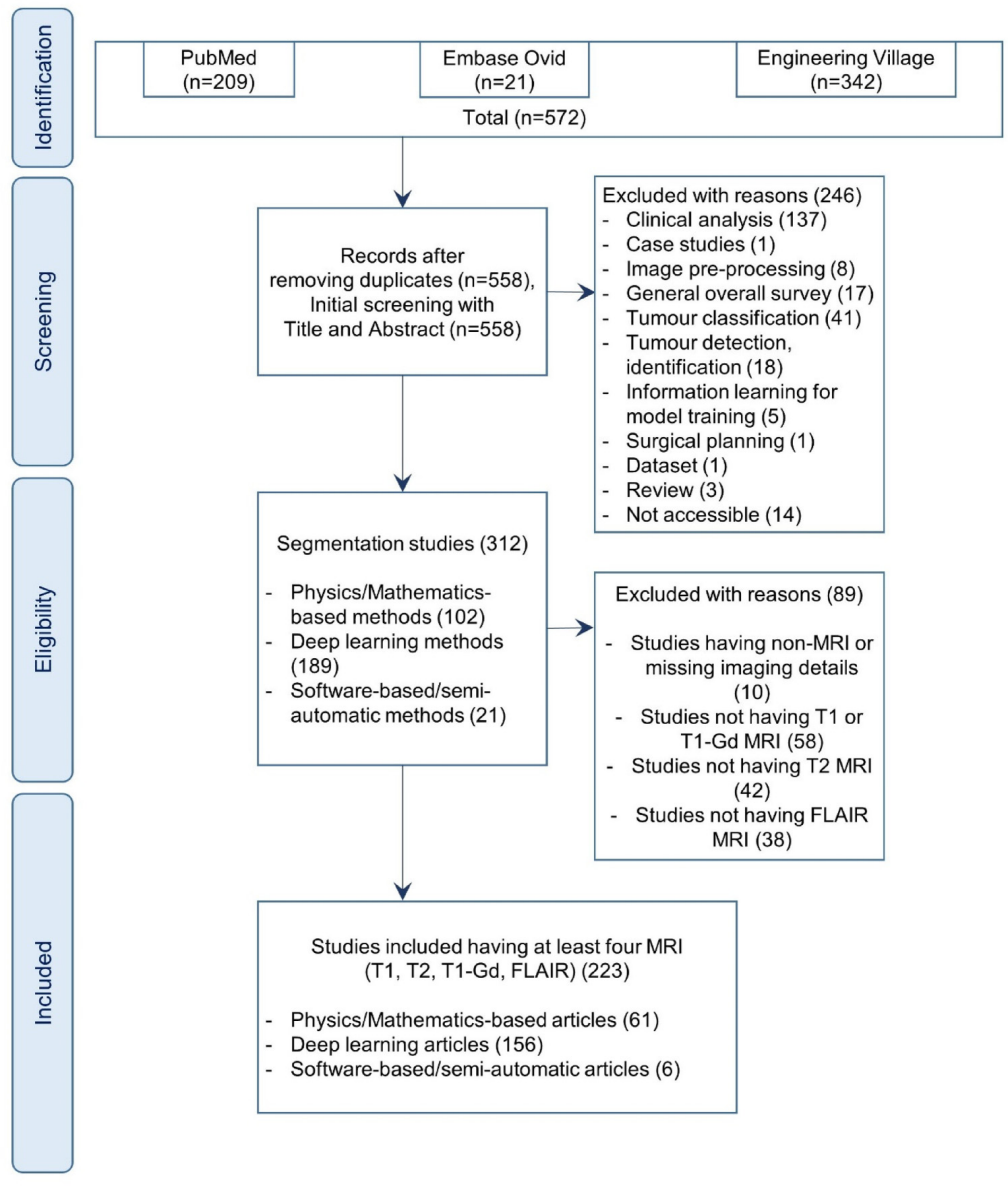
PRISMA diagram. PRISMA diagram of the systematic review of brain tumour segmentation methods.

Second, the segmentation studies are screened by reading the full text. At this level, the articles, which are eligible for synthesis, are identified by reviewing the imaging modalities, and associated data types used in their segmentation method. The studies, which are not MRI-based, are excluded. As a result, among the MRI-based studies, articles that utilised T1-weighted, T2-weighted, T1-Gd and FLAIR MRI are included for the final synthesis. All the inclusion/exclusion criteria are mentioned in the PRISMA^12^ diagram of Figure 1.

### Data extraction

After removing duplicates, all the articles are organised, as a table, in Microsoft Excel (Please refer to S1 Appendix). For each article, data is extracted according to the following parameters: publication id, publication type, publication year, author, article title, publisher, DOI, issue number, volume number, type of study, type of technical methodology, type of imaging modality, type of MRI, type of tumour, single or multi-centre data collection, the number of data samples and performance score.

Among the aforementioned parameters, the ‘data samples’ parameter is sub-categorised into study population, training data, validation data and test data. The types of technical methods in image-based tumour segmentation are organised into three categories: physics or mathematics-based methods, deep learning-based methods and software or semi-automatic methods. The performance score of each segmentation method is retrieved from the respective article and included in our table. In articles, authors have used various performance score measures, which include match, accuracy, Jaccard similarity coefficient, Tanimoto similarity, Hausdorff distance, dice score, positive predictive value, specificity, sensitivity, negative predictive value, precision, recall, misclassification error, intersection over union, Lin's concordance correlation coefficient, quality measure, balanced error rate, kappa, correlation, mean square error, false-positive rate per patient, the extent of resection, mutual information, residual tumour volume, root mean square error, the ratio of overlap, coefficient of variation, agreement index, interoperator variance, F1 score, volume difference, peak signal-to-noise ratio, qualitative evaluation, difference ratio of pixels, similarity, overlap index, absolute error, percent error, the difference with the gold standard, paired *t*-test mean difference, linear regression and area under the curve. The details of each performance score matrix can be found in their respective articles as mentioned in the data extraction matrix (S1 Appendix). We synthesised the performance of each method in segmenting whole tumour (WT), non-enhancing tumour core (TC) and enhanced tumour (ET). The values are stored as a tuple of ‘whole tumour, tumour core, enhanced tumour’. Each missing value in a performance score tuple is recorded as *null*. As shown in the PRISMA diagram of Figure 1, the imaging modalities, and MRI sequences are identified at the eligibility level. Broadly, four imaging modalities have been identified: magnetic resonance imaging (MRI), computed tomography, positron emission tomography, and ultrasound. Specifically in MRI, the identified imaging sequences include T1-weighted MRI, gadolinium-enhanced T1-weighted MRI (T1-Gd), T2-weighted MRI, FLAIR MRI, diffusion-weighted MRI, fMRI, perfusion-weighted MRI, magnetic resonance spectroscopy, apparent diffusion constant, fractional anisotropy, diffusion tensor, dynamic susceptibility contrast, dynamic contrast enhancement, diffusion kurtosis, magnetisation prepared rapid gradient echo, T1-MPRAGE and T1-weighted with turbo field echo.

### Risk of bias assessment

All reviewers assessed studies or validated data extraction matrix independently. JMB assessed each study and populated data extraction matrix records. SLCK validated the data extraction matrix. TNA verified, and confirmed the data extraction matrix. The whole process was performed manually, and without using any automation tools.

### Synthesis methods

Studies are synthesised according to the publication year, technical methods used in method development (type of study), MRI sequences used in the segmentation method development, deep learning methods (technical architectures used in deep learning studies), performance score to evaluate accuracy in segmenting three tumour components – whole tumour, and tumour core, and enhanced tumour, study population, and specific studies with additional MRI sequences. The studies having at least T1-weighted, T2-weighted, T1-Gd and FLAIR MRI sequences used are included for the synthesis. The synthesis of a number of articles is visualised as a bar plot, and pie charts showing the total number of articles in each year, and category. The synthesis of the study population in terms of data samples used, and performance score measure in terms of median Dice score are visualised as results. The outcome is depicted as Matlab box plots as commonly found in the synthesised articles. Deep learning studies are tabulated to identify the most widely adapted deep learning technology.

Benchmark review articles and particular imaging sequences studies are mentioned in the specific imaging studies’ section. Studies of segmentation methods using diffusion-weighted, and perfusion-weighted MRI, are reported individually.

During the synthesis, the unavailable values of performance score measures and data samples are considered as *null* values.

## Results

### Article identification

In this systematic review, 572 articles are identified from online publication repositories. Out of these, 14 duplicates are removed and the remaining 558 articles are screened for the eligibility criteria. After screening titles and abstracts, 246 articles are excluded, and the remaining 312 articles are screened by reading the full text. After the full-text screening, 89 articles are excluded with reasons, and 223 articles are included for synthesis. The summary of exclusion reasons and the number of excluded articles are shown in the PRISMA diagram (Figure 1).

### Publications over the time

**Figure 2.**
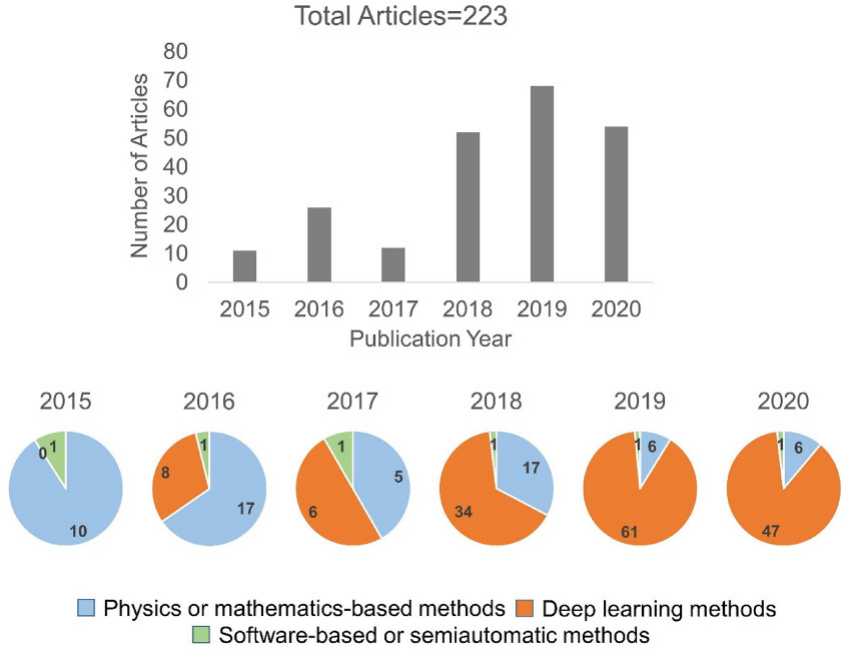
A number of articles (2015–2020). The bar plot represents the number of articles published over the review period (2015–2020), and pie charts depict published articles in each category of technical method in each corresponding year. Total articles = 223 refers to the articles included for the synthesis.

As shown in the PRISMA diagram, after screening at the abstract and title level, we selected 312 articles. Following that we applied the eligibility criteria, which provided us with 223 articles for the synthesis. These articles are categorised over the publication year, as shown in Figure 2 bar plot. Further, the articles are categorised as per the technical method category, as shown in Figure 2 pie charts.

### Type of study

After the screening at the title and abstract level, 312 studies are included. Among them, 102 are physics or mathematics-based, 189 are deep learning-based and 21 are software-based or semi-automatic methods articles. After the full-text screening, 223 studies are included for synthesis. Among them, 61 are physics or mathematics-based,^13–74^ 156 are deep learning-based and six are software-based or semi-automatic^75–80^ methods articles.

### Imaging sequences in synthesised studies

A total of 223 studies have used at least four MRI sequences (T1-weighted, T2-weighted, T1-Gd and FLAIR MRI) which are included in the final synthesis.

### Technical architectures in studies

We synthesised 156 deep learning articles to identify the commonly reported deep learning architecture in automatic brain tumour segmentation. We reported deep learning architectures such as convolutional neural network (CNN),^81–83^ visual geometry group (VGG) network,^84–86^ DeepMedic,^84,87–92^ U-Net,^84,91,93–114^ autoencoder,^115–117^ generative adversarial network (GAN),^118–120^ W-net,^113^ a cascade of W-Net E-Net, and T-Net,^113^ squeeze, and excitation network (SENet),^121^ multiresolution neural network,^122^ holistically-nested edge detection (HED) network,^123^ multi-level upsampling network,^124^ V-net,^125^ residual network (ResNet),^125,126^ hourglass network,^127^ multi-view network (MvNet),^128^ DeepSCAN,^129,130^ densely connected,^85^ inception,^85,99^ ensemble net,^131^ PixelNet,^132,133^ ContextNet,^134^ dense neural network,^135^ MC-Net,^136^ OM-Net,^136^ ConvNet,^137^ wide residual network, and pyramid pool network (WRN-PPNet),^138^ deep convolutional network,^139,140^ neuromorphic neural network,^141^ DeepLabv3 +,^142^ recurrent neural network^143^ and German cancer research centre (DFKZ) network.^107^ Moreover, we mentioned the extensions of these architectures in the discussion section. Deep architectures, and their extensions, with associated publications, are summarised in Table 1.

**Table 1.** Deep architectures, and their extensions used in tumour segmentation.

Deep architecture	Associated publications
CNN	^81–83,87,107,144–165^
VGG	^84–86,166,167^
DeepMedic	^84,87–92,168,169^
U-Net	^84,91,93,94,95,96,97,98–100,101–110,111–114,155,157,162,163,170,171,172–181,182–191,192–201,202–210^
Autoencoder	^115–117,211^
GAN	^118–120,212–214^
W-net and cascade of W-net E-net and T-net	^113,215–218^
SENet	^121^
Multiresolution neural network	^122^
HED	^123^
Multi-level upsampling network	^124^
V-net	^125,219^
ResNet	^125,126,197,220,221^
Hourglass network	^127^
MvNet	^128^
DeepSCAN	^129,130^
Densely connected	^85^
Inception	^85,99^
Ensemble net	^131^
PixelNet	^132,133^
ContextNet	^134^
Dense neural network	^135,222^
MC-Net	^136^
OM-Net	^136^
ConvNet	^137,223^
WRN-PPNet	^138,224^
Deep convolutional network	^139,140,159,225^
Neuromorphic neural network	^141^
DeepLabv3 +	^142^
Recurrent neural network	^143^
DFKZ	^107^
EMMA	^91^
SegNet	^226^
ResNeXt	^227^
DenseAFPNet	^228^
DMFNet	^229–231^
P-Net	^197^
MFNet	^232^
HNF-Net	^233^
Deep neural network	^234,235^
D2C2N	^236^
DeepSeg	^237^

CNN: convolutional neural network; VGG: visual geometry group; GAN: generative adversarial network; SENet: squeeze, and excitation network; HED: holistically-nested edge detection; ResNet: residual network; MvNet: multi-view network, WRN-PPNet: wide residual network, and pyramid pool network, DFKZ: German cancer research centre; EMMA: ensembles of multiple models, and architecture; DenseAFPNet: Dense atrous feature pyramid network; DMFNet: dilated multi-fibre network; MFNet: multi-direction fusion network; HNF-Net: high-resolution, and non-local feature network; D2C2N: dilated densely connected convolutional network.

### Performance score evaluation matrices of studies

**Figure 3.**
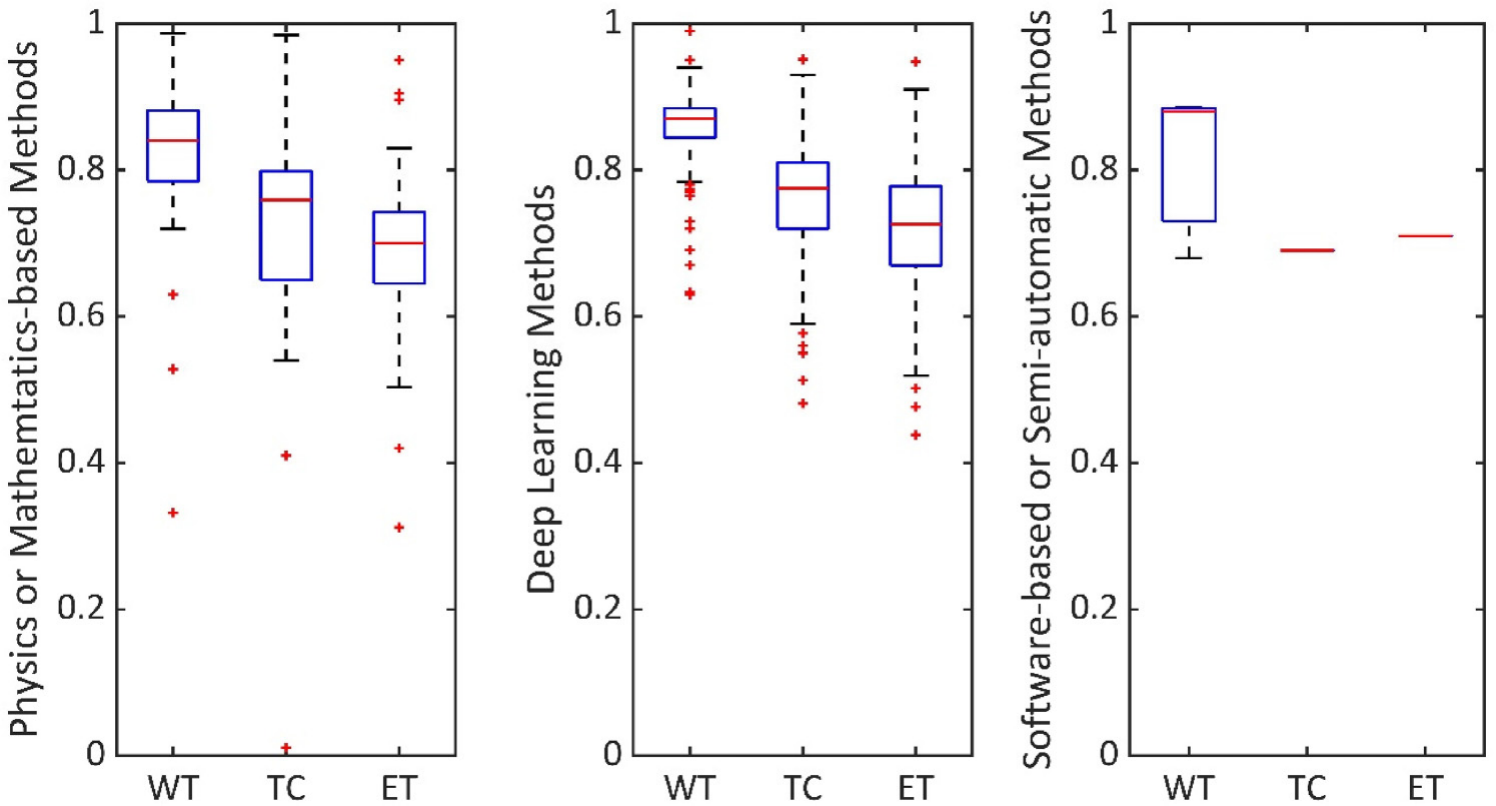
Comparison of segmentation results. Performance score evaluation, in segmenting WT, TC and ET, by considering all 223 articles.

From our data extraction matrix, it was apparent that the most common accuracy measure used is the Dice score. Therefore, in Figure 3, we have shown the median Dice score values in segmenting the WT, TC and ET areas of brain tumours, considering all the 223 articles. We have compared Dice score among physics or mathematics-based methods, deep learning methods and software-based or semi-automatic methods.

WT: whole tumour, TC: tumour core, ET: enhanced tumour.

### Study population

**Figure 4.**
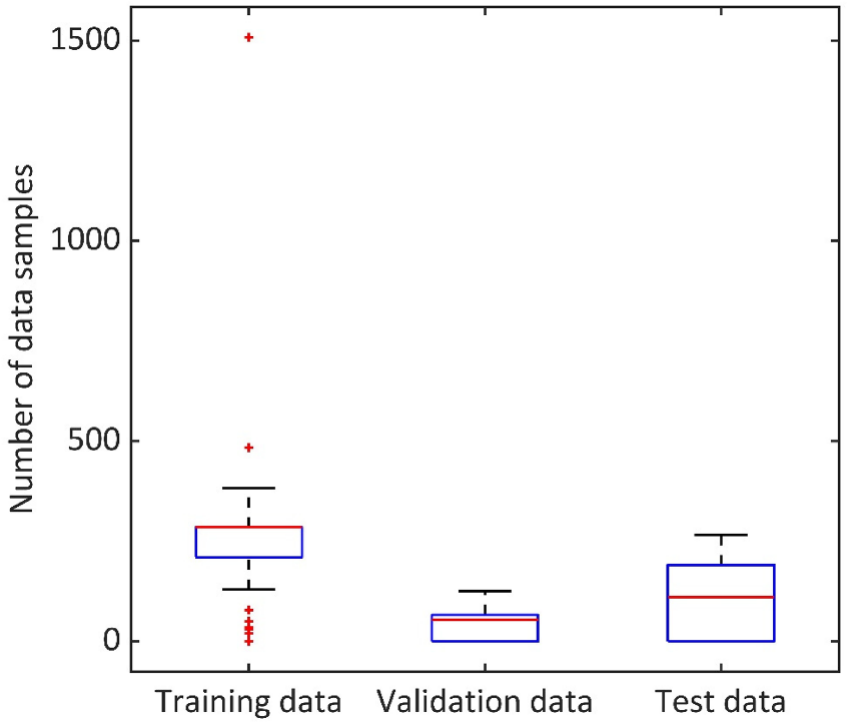
Data samples in deep learning studies. Summary of training, validation and test data samples reported in deep learning methods. Median of training, validation and test data samples are 285, 54 and 110, respectively.

The study population is summarised as the total number of data samples used in the study. The median data sample used in segmentation studies is 351 (median ± stdev = 351 ± 232.67). Moreover, deep learning segmentation methods are noted to have data samples divided into three categories: training data, validation data and test data. As shown in Figure 4, the median ± stdev of each training, validation and test data sample in deep learning methods are also reported, which is 285 ± 154.41, 54 ± 41.60 and 110 ± 85.29, respectively.

### Specific imaging studies

A rigorous review, using an open-access dataset, is published as the gliomas segmentation benchmark article,^5^ which encouraged a tumour segmentation method development using T1-weighted, T2-weighted, T1-Gd and FLAIR MRI sequences. Among the 223 synthesised articles, five studies have used additional imaging sequences. Three studies^71,117,236^ have used diffusion imaging sequences, and one study^212^ has used perfusion imaging and multiple CT imaging sequences. These three studies are noted to have deep learning, or physics or mathematics-based methods. Moreover, we noted that a few studies have used perfusion imaging sequences, and diffusion imaging sequences but did not use the aforementioned all four MRI sequences. These studies are perfusion imaging studies^212,238–241^ and diffusion imaging studies.^117,169,236,239–247^ Also, two studies are noted to have used ultrasound imaging.^248,249^

## Discussion

In this systematic review, we addressed a PICO style question to put the brain tumour segmentation methods in the context of clinical utility. Our population (P) is brain tumour patients, intervention (I) is segmentation methods, comparison (C) with manual segmentation evaluated as Dice score and outcome (O) is automated segmentation methods. Accordingly, we systematically found a promising technology, U-Net, which performs automated brain tumour segmentation using multiple MRI sequences. Its validation with manually segmented ground truth has achieved a Dice score of 0.9. Moreover, U-Net is majorly cited compared to other automated methods. Such merits show that U-Net has the potential to be considered for the clinical efficacy studies of automated brain tumour segmentation.[Table table3-20552076221074122]

**Table table3-20552076221074122:** 

Research in context
**Evidence before this study:** In the literature, individual studies of brain tumour segmentation methods, a benchmark framework (BraTS) to assess these methods using an open-access dataset of T1-weighted, T2-weighted, T1-Gd and FLAIR MRI are reported. A few studies used diffusion-weighted and perfusion-weighted MRI to validate their methods.
**Added value of this study:** U-Net is systematically identified as a promising technology (Dice score 0.9 and cited the most) for brain tumour segmentation. Moreover, it is noted that the contribution of open-access datasets, including diffusion-weighted, and perfusion-weighted MRI, should be encouraged for training, validation and testing of brain tumour segmentation algorithms.
**Implications of all the available evidence:** The identified technologies can help medical imaging specialists, and clinicians to semi-automatically or automatically segment brain tumours, compared to manually.

As shown in Figure 2 orange sections, deep learning articles in segmenting brain tumours are increasingly dominating the literature compared to software-based or semi-automatic methods, and considerably increased compared to physics or mathematics-based methods during the 2015–2020 period. The potential reasons could be the subjective nature of a software-based approach, limiting its efficiency, and limited transfer learning in physics-based models. Deep learning methods are attractive for efficient automatic segmentation, and the learned weights using one dataset can be further utilised with another dataset.

From a clinical point of view, the articles, which have reported segmentation of all three regions (whole tumour-WT, non-enhancing tumour core-TC, and enhanced tumour-ET), are synthesised. The whole tumour, including peritumoral oedema, is visible in FLAIR MRI, non-enhancing solid tumour core is visible in T2-weighted MRI, and necrotic/cystic core and enhanced core are visible in T1-weighted, and T1-Gd MRI.^5,250^ Specific details, such as cellular proliferation and cellular infiltration, can be visualised with diffusion-weighted imaging.^251,252^ Moreover, vascular proliferation can be visualised with perfusion-weighted imaging.^251^ The inclusion of tumour components, such as cellular proliferation, infiltration and vascular proliferation, are addressed in very few articles. The potential reason could be the limited availability of open-access diffusion and perfusion-weighted imaging datasets.

Segmentation methods are commonly validated in terms of Dice scores ranging from 0 to 1.0. 1.0 shows the best matching between segmentation ground truth and method output. As shown in Figure 3, physics or mathematics-based methods, deep learning methods and software-based or semi-automatic methods have median Dice score, as (WT, TC, ET) tuple, (0.84, 0.76, 0.7), (0.87, 0.78, 0.73) and (0.88, 0.69, 0.71), respectively. Particularly, physics or mathematics-based methods, and deep learning methods studies are enabled to explore accuracy in segmenting WT, TC and ET due to the availability of ground truth in datasets such as BraTS.^5^ As the whole tumour covers a larger area, the value of WT is higher compared to TC or ET in each category.

As shown in the PRISMA diagram Figure 1, we have synthesised articles with physics or mathematics-based methods, deep learning-based methods and software or semi-automatic methods. The technical architectures are specifically reviewed for deep learning-based methods, as they have the potential to transfer learning from one dataset to another dataset.^117^ Modifications of CNN are fully CNN,^81–83^ hyper-dense CNN,^161^ cascaded CNN,^162,163^ cascaded fully CNN,^164^ multi-path CNN,^87,165^ ensembled CNN,^144^ holistic CNN,^145^ full-resolution residual CNN,^146^ two-phase patch-based CNN^147^ and cascaded anisotropic CNN.^107^ U-Net is modified as cascaded U-Net,^162,163^ residual U-Net,^170,171^ domain adapted U-Net^172^ and efficient spatial pyramid (ESP) network.^173^ A modification of W-net is reported as the cascade of W-net, E-net and T-net,^113^ and a modification of V-net is reported as the cascaded V-net.^219^ Modifications of ResNet are dilated ResNet^220^ and fully convolutional residual neural network.^221^ An ensemble of multiple deep architectures is also reported as ensembles of multiple models, and architecture (EMMA).^91^ A modification of GAN is reported as conditional GAN.^212^ Modifications of ConvNet are reported as classification ConvNet^223^ and detection ConvNet.^223^

As summarised in Table 1, among all these deep architectures, U-Net based architectures are reported the most among the U-Net based articles, the maximum accuracy of segmenting a tuple of (WT, TC, ET) is reported as (0.92, 0.95, 0.94) in terms of Dice score. Several other architectures such as VGG, DeepMedic, autoencoder and GAN are also frequently reported, and extended. A summary of the technical specifications of U-Net, VGG, DeepMedic, autoencoder and GAN-based architectures is reported in Table 2. In physics or mathematics-based studies, and software-based or semi-automatic studies, the maximum reported Dice score in segmenting a tuple of (WT, TC, ET) is (0.97, 0.86, 0.95) and (0.88, 0.69, 0.71), respectively.

**Table 2.** Articles of widely used deep architectures and their technical details.

Deep architecture	Article	Technical details
U-Net	^84^	3D U-Net, which synthesises information at each scale by combining local and contextual information
	^103^	Modified 3D U-Net with better gradient flow
	^108^	Modified U-Net with an upsampled component, which is based on the nearest neighbour algorithm and elastic transformation
	^109^	2D U-Net network using a biophysics-based domain adaptation method with a generative adversarial model, which synthesises known ground truth data
	^110^	Modified U-Net with up skip connection, inception module and efficient cascade training
	^111^	3D U-Net with DenseNet, which was pre-trained on ImageNet
	^112^	U-Net network
	^113^	3D U-Net with test time augmentation
	^114^	U-Net with dice loss function to tackle class imbalance problem, and extensive data augmentation to prevent over-fitting
	^93^	Ensemble of 3D U-Nets with different hyperparameters
	^94^	U-Net training, which is followed by Bit-plane method output
	^95^	U-Net with double convolutional layers, inception modules and dense modules
	^96^	Modified U-Net, which is addressing class imbalance problem, with weighted cross-entropy and generalised dice loss function
	^97^	Deep learning radiomics algorithm model with 3D patch-based U-Net
	^98^	U-net with encoder adaption block and densely connected fusion blocks in the decoder
	^91^	An ensemble of two 3D U-Nets in which skip connections are used as a summation of signals in the up-sampling part of one network, and the other network uses concatenated skip connections and stridden convolutions
	^99^	Inception modules with U-Net
	^100^	Multi-scale images as input to the 3D U-Net and including 3D atrous spatial pyramid pooling layer to boost the performance of the network
	^101^	U-Net training improvement using large patch sizes, region-based training, additional data and a combination of loss functions
	^102^	U-Net with separable 3D convolution by dividing each 3D convolution block into three parallel branches
	^104^	Two 3D U-Nets in which the first detect the tumour, and the second one segments multiple regions of the tumour
	^105^	U-Net
	^106^	The tree structure of 3D U-Nets such that the first node of the tree predicts oedema, and then feed the output to the subsequent nodes to detect tumorous subregions of oedema
	^107^	U-Net in an ensemble of networks
VGG	^84^	3D fully connected network which is based on VGG with skip connections that combine coarse high scale information with fine low scale information
	^85^	3D convolutions, except max pool layers, VGG-based, an ensemble of multiple architectures
	^86^	CNN, which is based on VGG-16 and initially trained on ImageNet weights, and then fine-tuned with MICCAI data, relies on a pseudo-3D method which enables 3D segmentation from 2D colour-like images, and ultimately gives faster segmentation
DeepMedic	^84^	Two path network based on DeepMedic network which allows gathering low, and high resolution features together
	^87^	Multi-path CNN, which is inspired by DeepMedic which includes large, and small patches
	^88^	DeepMedic network
	^89^	A computer-aided diagnosis that combined DeepMedic, and radiomics features such as first-order features, shape features and texture features
	^90^	DeepMedic with additional residual connections
	^91^	An ensemble of two DeepMedic architectures
	^92^	A DeepMedic-based network is followed by a fully connected network to remove false positives
Autoencoder	^115^	Encoder, and decoder based 3D architecture that includes a variational auto-encoder branch to reconstruct the input image, which could be used as a regulariser for the shared decoder
	^116^	Stacked denoising auto-encoder
	^117^	Stacked denoising auto-encoder
GAN	^118^	Discriminator and generator based conditional generative adversarial network
	^119^	Adversarial network, discriminator is trained along with a generator to produce synthetic results, synthetic labels and ground truth are discriminated by discriminator, discriminator output is fed back to generator for improved segmentation accuracy
	^120^	Generative adversarial network with a coarse-to-fine generator to generate generic augmented data

VGG: visual geometry group; GAN: generative adversarial network; CNN: convolutional neural network; MICCAI: Medical Image Computing, and Computer-Assisted Interventions.

In this study, we have synthesised the study population and performance measures from the articles. Among the 223 synthesised articles, 217 studies have used multi-centre datasets, five studies have used single-centre datasets, and one study has used only a synthetic dataset. Note that, data variety could be limiting in the generalisation of Dice score performance comparison reported in our study. A potential solution is to develop an open-access data repository and review the studies with the same data samples. Moreover, some of the studies have missing values, which could limit our synthesis results. A benchmark framework to report the evaluation measures could be useful to mitigate such limitations in the future. In this study, we identified brain tumour segmentation techniques and synthesised results as found from their respective articles, which could be a limitation. In order to apply these methods at clinics, a separate efficacy study should be performed by clinical staff members utilising data at respective clinics.

## Conclusion

In conclusion, we systematically addressed a review question that can help medical imaging specialists, and clinicians to identify automatic brain tumour segmentation techniques, compared to manual segmentation. Our specific inclusion criteria emphasised having multiple MRI sequences in the method development. We noted that four MR-based sequences, i.e. T1-weighted, T2-weighted, T1-Gd and FLAIR MRI, are used the most. Diffusion weighted, and perfusion-weighted MRIs are rarely used. Among the segmentation methods, deep learning methods have contributed the most compared to other methods during the 2015–2020 period. Within the deep learning methods, U-Net-based methods are adapted the most and have an accuracy of approximately 0.9 Dice score in segmenting a brain tumour. We also noticed that the benchmark BraTS dataset does not have perfusion-weighted and diffusion-weighted MRI data, motivating the development of an open-access data repository with such MRI sequences.

In the future, a novel dataset can be developed with additional imaging data such as diffusion-weighted, and perfusion-weighted MRI, similar to the frequently reported open-access dataset^5,6^ which contains the data samples of T1-weighted, T2-weighted, T1-Gd and FLAIR MRI of gliomas patients. Creating such an open-access dataset can help to include cellular proliferation, infiltration and vascular proliferation in brain tumour segmentation techniques (cellular proliferation: increase of the number of cells; cellular infiltration: migration of cells or excessive growth; vascular proliferation: leaky blood vessels). Moreover, medulloblastoma cases are rare (European annual rate: 6.8/million, age: 0–14 years, duration: 2000–2007).^253,254^ Therefore, a comprehensive adult dataset may be useful to address childhood tumours with transfer learning methods.

## Supplemental Material

sj-xlsx-1-dhj-10.1177_20552076221074122 - Supplemental material for Magnetic resonance image-based brain tumour segmentation methods: A systematic review Click here for additional data file. Supplemental material, sj-xlsx-1-dhj-10.1177_20552076221074122 for Magnetic resonance image-based brain tumour segmentation methods: A systematic review by Jayendra M Bhalodiya, Sarah N Lim Choi Keung and Theodoros N Arvanitis in Digital Health
